# Sirtuins (SIRTs) As a Novel Target in Gastric Cancer

**DOI:** 10.3390/ijms232315119

**Published:** 2022-12-01

**Authors:** Agata Poniewierska-Baran, Paulina Warias, Katarzyna Zgutka

**Affiliations:** 1 Institute of Biology, University of Szczecin, Felczaka 3c, 71-412 Szczecin, Poland; 2 Department of Physiology, Pomeranian Medical University in Szczecin, Powstancow Wielkopolskich 72, 70-111 Szczecin, Poland; 3 Department of Physiology in Health Sciences, Faculty of Health Sciences, Pomeranian Medical University, Szczecin, Żołnierska 54, 70-210 Szczecin, Poland

**Keywords:** sirtuins, SIRT family, gastric cancer, stomach cancer, gastric adenocarcinoma, pathogenesis

## Abstract

Gastric cancer is a major health burden worldwide. Among all neoplasms, gastric cancer is the fifth most common and the third most deadly type of cancer. It is known that sirtuins (SIRTs), are NAD^+^-dependent histone deacetylases regulating important metabolic pathways. High expression of SIRTs in the human body can regulate metabolic processes; they prevent inflammation but also resist cell death and aging processes. The seven members of this family enzymes can also play a fundamental role in process of carcinogenesis by influencing cell viability, apoptosis and metastasis. This review collects and discusses the role of all seven sirtuins (SIRT1–SIRT7) in the pathogenesis of gastric cancer (GC).

## 1. Introduction

Gastric cancer (GC) remains a serious clinical problem and a challenge for doctors and scientists. Early-stage GC is usually asymptomatic or causes non-specific symptoms, such as feeling full after eating a small amount of food, heartburn, nausea, stomach pain or unintentional weight loss [1]. Conventional therapies for advanced GC include surgery, chemotherapy and radiotherapy, and the length or quality of life of patients with advanced GC remains unsatisfactory. One of the causes of GC is dietary habit [2] and infection caused by *Helicobacter pylori* (*H. pylori*), which causes the development of local inflammation [3]. The activation of immune responses due to the presence of pathogens involves the mobilization of cells—phagocytic neutrophils and macrophages. The infiltration of phagocytic cells leads to the production of superoxide (O_2_^⨪^) and nitric oxide (NO) to kill invading microbes in phagocytes [4]. ROS (reactive oxygen species) -mediated stress responses result in gastric mucosal injury, ulcers, and ultimately the development of GC [5]. This causes the release of large amounts of cytokines in tissue, such as interleukin 1 and 6 (IL-1, IL-6) and tumor necrosis factor-α (TNF-α), which initiate the acute phase inflammatory response, and has metabolic consequences [6]. 

The tumor microenvironment (TME) is crucial for tumor development, progression and response to selected therapy [7]. We can say that cancer is a natural consequence to an abnormal stromal environment [8]. Due to the fact that carcinogenesis stimulates TME cells to proliferate and secrete locally acting cytokines, it shows some similarities to the wound healing process, hence the statement that tumors can be perceived as “non-healing wounds” [9,10]. The tumor microenvironment is made up of many components, such as normal endothelial cells (EC), fibroblasts (Fb), pericytes (PC) and inflammatory cells (e.g., T cells and macrophages), but TME also includes special cells with changed phenotypes, characteristic for the area of tumor formation and progression, such as tumor-associated macrophages (TAM) and cancer-associated fibroblasts (CAF). These cells can secrete pro-inflammatory molecules, such as interleukins (e.g., IL-6), chemokines (e.g., CXCL12/SDF-1α), vascular endothelial growth factors (VEGF), platelet-derived growth factors (PDGF), matrix metalloproteinases (MMP), and components of the extracellular matrix (ECM; e.g., tenascin C, fibronectin, and collagen type I) [11]. These molecules and cytokines recruit bone marrow-derived cells and immune cells into their vicinity. In the inflammatory microenvironment of the tumor, energy metabolism is reorganized and, importantly, many sensors of energy metabolism have an immunoregulatory effect [12]. 

Research on the sirtuin protein was started in 1991 by Leonard Guarente, and the first sirtuin was identified in yeast (sir2). After discovering that enzymatic proteins control the cell metabolism, the role of seven sirtuins in many age-related diseases were evaluated [13,14], such as diabetes type 2 [15], cardiovascular diseases [16], RA [17] or cancers [18]. Indeed, SIRTs are the key regulators of clinically significant cellular processes that can play an important role in cancer. They regulate chromatin, metabolic homeostasis, development, differentiation, and the survival of cells [19]. Sirtuins are a large family with seven members (SIRT1–7), and their role in the carcinogenesis is openly discussed. In this review we will present numerous examples showing that sirtuins play a crucial role in gastric cancer, acting either as a tumor suppressor or as an oncogenic factor, or even acting in a dual role—like SIRT1. The particular challenges include understanding and detailing the mechanism of Sirtuins activation and deactivation in gastric cancer. Most reports and studies are based on SIRT1 in GC, but the functions of the six other sirtuins are now emerging, including curiosity—do other sirtuins also have this dual role (like SIRT1), or can they perhaps switch from cancer promotor to suppressor? More and more questions arise.

## 2. Gastric Cancer

A gastric cancer tumor is formed from neuroendocrine cells, gastric mucosa, and connective tissue of the stomach walls or lymphatic tissue [20]. GC metastasizes from the stomach to the lungs, bones, the liver and lymph nodes [21]. Its progression and development is a process that involves a range of environmental and genetic factors. Over one million new cases of GC were diagnosed in 2018, which makes it the fifth most common cancer worldwide. An estimated 783,000 people worldwide died of GC in 2018. These figures make GC the third most deadly type of cancer [22]. Adenocarcinoma is the most common kind of stomach malignant cancer that makes up 90–97% of all malignant gastric tumors [23].

The Lauren classification, which takes cell morphology and type of neoplastic infiltration into consideration, divides GC into two major subtypes; intestinal and diffuse. The classification is useful in estimating prognosis and making clinical decisions. The intestinal type is morphologically similar to intestinal mucosa. It has mostly a glandular structure, with cells similar to intestinal cylindrical cells and with goblet cells that produce sour mucopolysaccharides. The intestinal type is usually accompanied by atrophic gastritis with intestinal metaplasia. It has a better prognosis. A recent decrease in GC incidence was mainly observed in the intestinal type. The diffuse type forms single cells or small groups of cells with little adhesion. It is characterized by intramural infiltration of scattered tumor cells that do not create clear boundaries. Due to its high malignancy, it has a worse prognosis.

The most common causes of GC are age [24], gastroesophageal reflux disease [25], infection by *H. pylori* bacteria, [3] and off course diet [2,26]. Dietary habits are one of the causes of increased carcinogenesis in developing societies. Eating hot meals, salted, fried, pickled or smoked foods, as well as nitrogenous substances and aromatic hydrocarbons are important causative factors in stomach cancer. It turned out that the introduction of vegetables and fruits to the diet can reduce the risk of developing the disease by up to 66–75% [26]. The number of GC incidence has decreased recently, but most diagnoses are made in advanced stages with poor prognosis. The incidence of GC is tightly correlated with environmental factors and geographical location. Approximately half of GC cases are diagnosed in East Asia. 

The main therapy in gastric cancer is the surgical removal of pathologically changed tissues with a safety margin of healthy tissue; however the 5-year survival rate for patients has not satisfactorily increased. Preoperative and adjuvant treatment (chemotherapy, radiotherapy and chemoradiotherapy), are now the gold standard in many countries, as a first step before the main treatment (surgery), or as an adjunct to surgery [27]. Both methods increase patient survival. Improvements in surgical techniques and advances in traditional radiotherapy, chemotherapy and neoadjuvant therapy have increased the 5-year survival in early gastric cancer to >95%. The problem remains the low rate of early diagnosis (the best surgical window is missed), meaning that patients are diagnosed more frequently at an advanced stage. The main treatment for advanced gastric cancer remains a combination of neoadjuvant chemoradiotherapy, molecular-targeted therapy, and immunotherapy [28]. Chemotherapy is mainly based mainly in inter alia infusions with epirubicin, cisplatin, mitomycin (FAM) or 5-fluororuracil [27,28].

Unfortunately, the median of overall survival (OS) for GC patients is still unsatisfactorily low. Gastric cancer remains a burden for societies, therefore further research into the treatment, GC risk factors and new therapeutic goals is essential [29].

## 3. Sirtuins (SIRTs) Family

**Figure 1:**
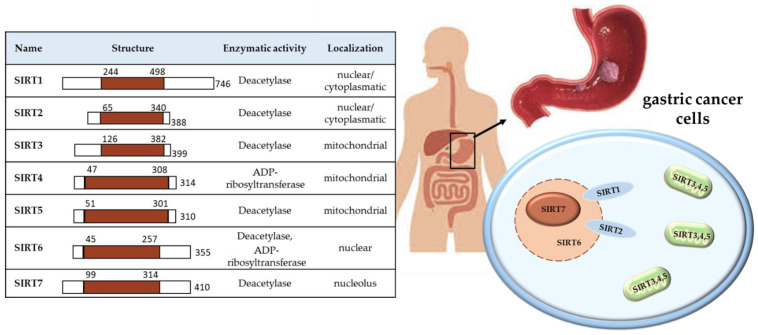
Characteristics of the SIRTs location and role in the cell.

There are seven sirtuins in mammals (SIRT1–7), which are NAD^+^-dependent deacetylase proteins. They have been confirmed for a variety of phylogenetic species, from archaea to human. Their structure, activity and location in the cell are specific and different between individual SIRT. Sirtuin 1 (SIRT1) and sirtuin 2 (SIRT2) are located in the nucleus and/or cytoplasm, with the possibility of circulation between them; SIRT3–5 in mitochondria, while SIRT6 in nucleus and SIRT7 in nucleolus, as shown in Figure 1.

Sirtuins are mainly involved in the metabolic regulation of cells, but also in some biological processes, such as cell survival [30], apoptosis [31], autophagy [32], proliferation [33], cellular senescence [34], stress response [35], genome stability [36], etc. Many cellular processes are regulated by deacetylation reactions, and some members of the histone deacetylases (HDACs) classes (e.g., sirtuins) can be overexpressed in cancers [37]. It is believed that abnormal patterns of protein acetylation may promote neoplastic transformation and tumor progression [37]. Yamamoto et al. [38] summed up the participation of the SIRTs in the endocrine signaling, glucose homeostasis, even aging and longevity [39,40,41,42]. It is known that cancer cells and the surrounding TEM cells interact with each other to change their metabolism. The most striking feature of metabolism reprogramming of tumor cells is the “Warburg effect”—cancer cells are more dependent on aerobic glycolysis than the mitochondrial oxidative phosphorylation system—OXPHOS [43]. Glycolysis could provide a rapid energy supply and several beneficial conditions for the genesis and progress of the tumor microenvironment. To meet the needs of continued growth and proliferation, tumor cells provide themselves with more material and energy through metabolism reprogramming [43]. Many clinical observations and metabolomic analyses have revealed metabolic complications in age-related diseases, cancers, as well as in autoimmune diseases (Ad) [44,45,46]. Most of the well-characterized oncogenes or tumor suppressor genes function to sustain the altered metabolic state in cancer. It has been shown that sirtuins have an important role in regulating immunometabolism in immune cells—the most widely studied are in macrophages and DCs [47]. SIRT suppresses both innate and adaptive immunity. Increased glycolysis during the early inflammatory phase results in the high concentration of NAD^+^, which induces SIRT6 and SIRT1 activity. SIRT6 inhibits glucose metabolism, while SIRT1 promotes FAO through the activation of transcriptional coactivator PGC1α. It has been demonstrated that SIRT1 can inhibit NF-κB signaling, by deacetylate the NF-κB subunit (p65), and promotes oxidative metabolism associated with the resolution of inflammation [48]. The myeloid deletion of SIRT1 causes the hyperacetylation of NF-κB in bone marrow-derived macrophages (BMDMs), increasing transcriptional activity and the secretion of TNFα and IL-1β as the pro-inflammatory cytokines [49]. Depending on the stimuli received, DCs release a different pattern of cytokines that will drive the differentiation of T helper (Th) cells or T regulatory (Treg) cells. SIRT also affect T cells as shown [50]; more precisely, SIRT1 promotes Th2 responses through the inhibition of peroxisome proliferator-activated receptor-γ (PPARγ) activity and SIRT1 expression is highly induced in anergic T cells. In regulatory T cells (Treg), SIRT1 deacetylate FOXP3, leading to the degradation of FOXP3 through proteosomes, and TNF-α increases the pro-inflammatory factors—MMP-9, IL-1β, IL-6 and iNOS. 

SIRTs can affect immune cell receptors and immune response to modulate the development of chronic autoimmune and/or inflammatory diseases. All of this clearly indicates that the participation of SIRTs in the pathogenesis of neoplasms, including GC, must be significant.

## 4. Role of Sirtuins (SIRTs) in Progression and Metastasis of GC Cells

The metastasis of GC is the process of spreading cells from a primary tumor to a different part of the body, and is responsible for the majority of cancer-related deaths. Stopping this process is a challenge for scientists around the world, and the most important is to understand the mechanisms involved in cancer migration and invasion. Many studies have confirmed the functions of SIRTs in the migration and formation of neoplastic metastases, in various types of cancer [51], such as breast cancer [52], colorectal cancer [53], esophageal squamous cell carcinoma [54], hepatocellular cancer [55], ovarian carcinoma [56], pancreatic ductal cancer [57], non-small cell lung carcinoma [58], leukemia [59], prostate cancer [60], as well as gastric cancer [61,62,63,64,65], and others.

The influence of SIRTs on the GC progression and metastasis does not start when the primary tumor arises and grows, or even when its formation begins. It has been found that SIRTs participates in the development of the inflammatory process, caused by *H. pylori* infection, by increasing the proinflammatory cytokine gene expression in gastric epithelial cells. SIRT1 and SIRT2 as deacetylases play a certain role in the progress of inflammation by activation NF-κB signaling. It was also shown that *H. pylori* infection increases SIRT2 gene expression in gastric epithelial cells of gastritis patients [66,67]. This suggests that SIRTs may be a key modulator in the GC pathophysiology from its earliest stages.

**Figure 2:**
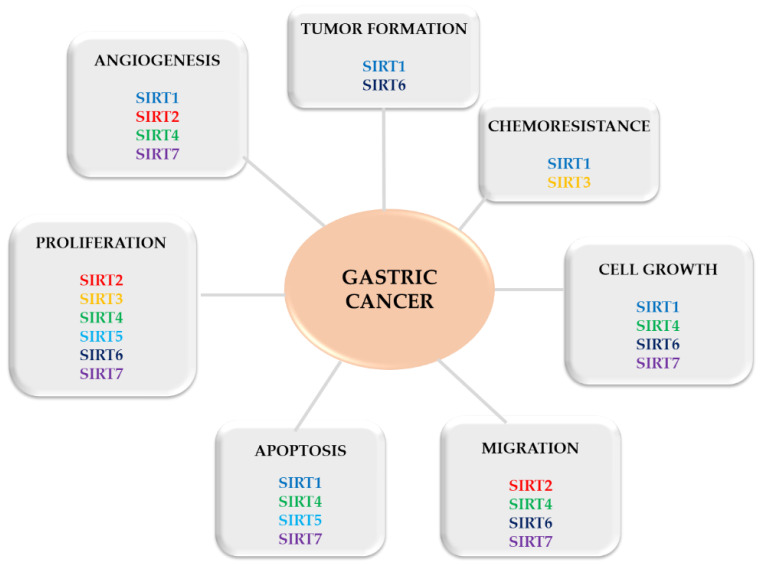
Progression of gastric cancer controlled by SIRTs.

Several studies have reported that SIRT (e.g., SIRT1) plays a role in the invasion, epithelial-mesenchymal transition and even chemoresistance in GC cells [68,69], becoming an important target for treatment. Interestingly, sirtuins seem to play a double role in cancer, acting as a tumor suppressor or promoter, affecting all stages of tumor development (Figure 2). By increasing genomic stability and limiting cell replication [70], they protect the organism against neoplasms, but can also induce oncogenesis by promoting cell survival in stress conditions [30] and by improving uncontrolled cell division [64,71]. This double-face character of sirtuins in cancer (as a promotor or suppressor) can be linked to their key role in cellular pathways, such as cell growth, cell cycle, genome integrity and stress-induced cell death [64]. To explain this dual-role, some researchers associated the SIRT with the presence (or not) of p53—a regulatory protein that is often mutated in human cancers [72]. Others explained that it may be determined by SIRTs’ subcellular localization [73].

The influence of the SIRTs and their role in GC are summarized in Table 1 and detailed below in Section 4.1, Section 4.2, Section 4.3, Section 4.4, Section 4.5 Section 4.6 and Section 4.7.

### 4.1. Sirtuin 1

The Sirtuin 1 (SIRT1) gene is the most frequently studied family member of all SIRTs in various types of cancer, including GC. SIRT1 is an enzyme located in the cell nucleus, but it can move to the cytosol via two nuclear localization sequences (NLS) and nuclear export signals (NES) [85]. SIRT1 expression depends on tumor type, microenvironmental complexity and the effects of cellular stress, such as caloric restriction, starvation conditions (fasting) or the presence of ROS. 

There are numerous studies reporting high expression levels of SIRT1 in cancers, such as breast cancer [86], colorectal adenocarcinoma [87], hepatocellular carcinoma [88], soft tissue sarcomas [89], prostate cancer [90], ovarian and cervical cancers [91], lymphoma [92], and GC [77,93]. Interestingly, the low expression of SIRT1 has also been reported also colorectal cancer [94,95] and GC [96]. In all cases, SIRT1 has been described as a good prognosis indicator for disease progression [97].

The diverse location of SIRT1 and its constant shuttling between cytoplasm and nuclei enables deacetylation of histone substrates, but also transcription factors and cofactors—p53, STAT3, DBC1, FOXO, c-Myc, and Ku70. Zhang et al. [75] suggest to call SIRT1 as an early diagnostic and prognostic marker of GC. Zhang’s team demonstrated the increased expression of SIRT1 in all examined cancer stages compared to mucosa without neoplasm. They also found a significant correlation between SIRT1 gene expression and the stage of GC and between advancement stage of gastric adenocarcinoma and poor prognosis. They showed that SIRT1 is involved in the mitophagy necessary for maintaining neoplastic homeostasis. Yap (Yes-associated protein)—induced SIRT1 gene activates mitofusin 2, which is currently considered to be the main controller of mitophagy. This pathway blocks the apoptosis track that involves caspase 9 and reduces the oxidative stress of tumor cells. Consequently, the expression of F-actin necessary for the production of lamelliopodia is increased. The increased migration of cancer cells ultimately follows [98].

Interestingly, there is evidence that the SIRT1 gene seems to inhibit GC cell growth. Lu et al. [99] observed that the activation of SIRT1 by resveratrol counteracted the activation of STAT3 (signal transducer and activator of transcription), and NF-κB. STAT3 plays an important role in mediating extracellular signals and it regulates the transcription of genes responsible for angiogenesis, cell proliferation, and survival. The blockage of STAT3 signaling pathway provides an attractive strategy for therapeutic intervention and is a potential method of increasing cancer treatment response [100]. Another element involved in the oncogenesis is p53—a transcription factor with tumor suppression properties. It is the first identified non-histone target of SIRT1. The p53 protein can be either deacetylated or destabilized by SIRT1, which inhibits its ability to halt the cell cycle and apoptosis. It was demonstrated that inhibition of SIRT1 activity with Tenovine 6 led to p53 activation and reduced tumor growth. A similar observation was made after the application of DBC1 (deleted in breast cancer-1), SIRT1′s negative regulator. This factor binds to the catalytic domain of SIRT1 and inhibits its deacetylase activity, which in turn facilitates p53 hyperacetylation and boosts apoptosis [77].

SIRT1 in patients with gastric adenocarcinoma was demonstrated to be able to play the role of cancer suppressor through the interaction with β-catenin, which is a significant regulator of the Wnt signaling pathway, which is important in cell adhesion. SIRT1 inhibits β-catenin location in the nucleus and significantly weakens its ability to activate transcription. This hypothesis can explain how the expression of SIRT1 and cytoplasmic β-catenin is correlated in patients with gastric adenocarcinoma and how patients with SIRT1 and β-catenin have better survival rates. A similar finding was observed in the same study. The combined expression of both DBC1 and SIRT1 significantly correlated with the high expression of cytoplasmic β-catenin and a better survival rate [101].

**Figure 3:**
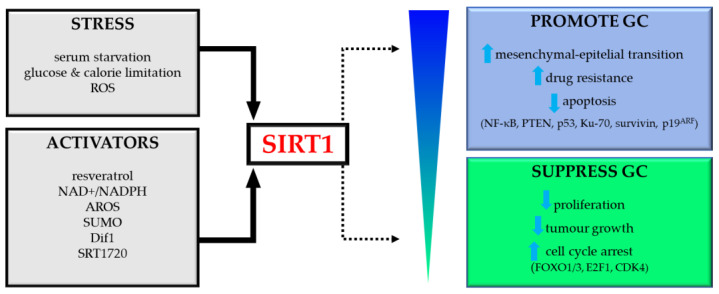
Dual role of SIRT1 in gastric cancer (GC). Abbreviations: ROS, reactive oxygen species; AROS, active regulator of SIRT1; SUMO, small ubiquitin-like modifier; PTEN, phosphatase and tensin homolog deleted on chromosome ten; CDK4, cyclin-dependent kinase 4.

Future research should focus on elucidating the molecular pathways and targets controlled by SIRT1. This may provide a more precise and effective treatment tool in GC, limiting the adverse effects of therapy. The role of SIRT1 in GC has been schematically shown in Figure 3.

As already mentioned in this work, SIRT1 can be a tumor promoter or a tumor suppressor.

Previous studies have shown that SIRT1 is downregulated in GC and inhibits GC cell proliferation and xenografted tumor growth [64]. They provide evidence that SIRT1 acts as a critical negative regulator of the metastasis of GC, and a higher expression of SIRT1 correlates with longer overall survival. After in vitro and in vivo experiments, they postulated that SIRT1 suppressed the migration and invasion of GC cells and limited lung metastasis of GC. To confirm, data from the Kaplan–Meier database show that increased SIRT1 levels result in better overall survival [64]. In fact, SIRT1 expression is negatively correlated with tumor TNM stage and lymphatic invasion [100]. SIRT1 can also inhibit GC by repressing cell proliferation and thereby tumor growth, or by inducing a G1-phase cell-cycle arrest and cell aging by FOXO1/3, E2F1 and CDK4 [97].

SIRT1 can also act as a promoter in GC. Cha et al. [77] showed that nuclear expression of SIRT1 was detected in 73% of GC patients and correlated with tumor invasion (tumor stage and metastasis to lymph nodes). In a GC mouse model study, it was shown that the expression of the SIRT1 protein was significantly higher in obese mice with GC than in lean mice with GC. This is a very interesting observation and confirms that caloric restriction and/or starvations effect on SIRT1 expression and, thus, the development of GC [102].

SIRT1 can be modulated by intracellular and environmental factors, such as cellular stress (starvation, glucose and calorie restriction); some protein factors (AROS, SUMO, NAD+/NADH, HuR, DBC1and Dif1), by natural and synthetic agonists (resveratrol, SRT1720); or by inhibitors (tenovins, sirtinol) [97]. Detailed methods of regulating and modifying SIRTs with natural or synthetic activators and inhibitors are presented in the last section of this review and are summarized in Table 2.

### 4.2. Sirtuin 2

Sirtuin 2 (SIRT2) is located in the cell nucleus and cytosol. Recent research has cast new light on SIRT2 as a pro-metastasis factor that promotes GC cell migration and invasion [62]. The increased expression of SIRT2 was observed in material obtained from 84 patients with GC, and correlated with poor prognosis and shorter survival time—OS. The GC cell lines AGS, HGC-27, MGC-803 and MKN-45 also had increased SIRT2 expression compared to GES-1 control line [62]. Although a CCK8 and colony formation assay showed that the overexpression of SIRT2 marginally promoted the proliferation in GC cell lines, SIRT2 knockdown or the use of SirReal2 selective inhibitor decreased the migration and invasion of GC cells.

### 4.3. Sirtuin 3

Sirtuin 3 (SIRT) is located in the mitochondrial matrix, and plays an important role in regulating mitochondrial metabolism, including amino acid metabolism, fatty acid oxidation, the tricarboxylic acid cycle, and the urea cycle. SIRT3, as well as SIRT1, is a double character enzyme, and can be a promotor and suppressor of GC biological processes. The first findings suggested that SIRT3 was the inhibitor. GC patients had reduced SIRT3 expression and it had reverse correlation with changes observed clinically, including tumor differentiation, stage and infiltration [103]. SIRT3 was supposed to be the main biomarker of better prognosis in GC. Wang et al. observed that the overexpression of SIRT3 in AGS, SGC-7901 and BGC-823 GC lines significantly suppressed the level of proliferation and colony formation. They stated that on the molecular level, SIRT3 inhibited the expression of Notch-1 both at the mRNA and protein levels. Similarly, SIRT3 knockdown promoted cell division and thus tumor growth [78].

In a study conducted by Lee et al. [79], the infection with *H. pylori* was demonstrated to lead to degradation of mitochondrial SIRT3. In GC lines, the expression of this deacetylase suppressed tumor growth by the reduction of HIF-1α and reduced the production of reactive oxygen species (ROS). The lines infected with *H. pyroli* exhibited increased activity of HIF-1α and ROS production. The level and activity of SIRT3 decreased depending on oncoprotein CagA (cytotoxin associated protein A). However, another study [80] showed that SIRT3 expression promoted the proliferation and increased generation of ATP, glucose uptake, activity of manganese superoxide dismutase (MnSOD), and lactate production in GC cells. SIRT3 gene knockdown inhibited these effects. These observations point out the role of SIRT3 in processes involving the reprograming of GC cell bioenergy. Interestingly, patients with intestinal GC were characterized with increased SIRT3 expression compared with the diffuse type of GC. 

SIRT3 was also demonstrated to cause the deacetylation of lactate dehydrogenase A (LDHA), thus boosting its activity. LDHA is the main protein that regulates glycolysis, which is instrumental for tumor growth. Increased LDHA levels in GC patients correlate with better response to chemotherapy and unfortunately also with shorter overall survival [81]. Recent research showed that genetic polymorphisms of mitochondrial sirtuins, including SIRT3, are linked to GC pathogenesis [104]. Changes that involve these genes lead to mitochondrial dysfunctions, thus promoting carcinogenesis. Further research is necessary to determine whether or not some single-nucleotide polymorphisms (SNPs) can be used as prognostic markers of GC. 

### 4.4. Sirtuin 4

Sirtuin 4 (SIRT4) is a mitochondrial enzymatic protein that exhibits NAD^+^- dependent deacetylase activity, thus performing an important function in human inflammatory diseases. Human SIRT4 is to be found mainly in the mitochondria and has many targets, including glutamate dehydrogenase, malonyl CoA decarboxylase, pyruvate dehydrogenase, ADP-ribose, deacetylan, deacylan, or depolypolian, which mitigate key metabolic pathways. SIRT4 was also demonstrated to be downregulated in GC human samples. In cell models, SIRT4 genetic damage induced cell growth and MKN-45 and HGC-27 GC cell invasion [105]. 

Hu et al. [82] conducted a study on SGC-7901 and MNK45 human GC cell lines infected with a vector which induces SIRT4 overexpression. The study showed the significant inhibition of human GC cell proliferation. The colony formation test showed that SIRT4 overexpression significantly reduced the number of colonies formed in vitro by SGC-7901 and MNK45 cells. Flow cytometry showed the inhibition of GC cell proliferation. SIRT4 overexpression induces the stoppage of the GC cell cycle and increases the number of cells during G1, which results in a smaller number of cells in the S phase compared to controls. SIRT4 significantly increased the share of SGC-7901 cells in G2. Research confirmed that SIRT4 overexpression did affect GC cell apoptosis [82]. 

Another study by Huang et al. [106] determined the level of SIRT4 expression in gastric adenocarcinoma and equivalent healthy stomach tissue with immunohistochemical staining. SIRT4 expression in gastric adenocarcinoma was substantially lower than that in healthy tissue. Additionally, low SIRT4 correlated with higher malignancy of GC, tumor growth (infiltration depth), metastases to successive lymph nodes, and a higher number in UICC classification of cancer staging. These findings suggest the suppressive activity of SIRT4 towards cancer [106]. 

Sun et al. [65] show that SIRT4 acts as a GC tumor suppressor by reducing cell proliferation. The expression of SIRT4 was downregulated in GC tissues as well as GC cells. Cell migration and invasion was also controlled by SIRT4, thereby affecting the epithelial-mesenchymal transition.

### 4.5. Sirtuin 5

Sirtuin 5 (SIRT5) is also a mitochondrial enzymatic protein, regulates cellular metabolism by participating in the β-oxidation of fatty acids, the tricarboxylic acid (TCA) cycle, and glycolysis [107]. The SIRT5 function in humans is to remove acyl groups, such as glutarate, malonate and succinate from lysine residues in different mitochondrial enzymes. It was found to modify proteins involved in the urea cycle, glycolysis, beta-oxidation and ketogenesis. It was also demonstrated that these post-translational modifications have pro- or anti-cancer activity, depending on cancer type and cancer location [108]. The study was conducted on GC cell lines (MKN7, AGS, SUN1 and HCG27) which were infected using vectors with SIRT5 overexpression. SIRT5 expression was substantially reduced in most tumor cells compared to adjacent healthy tissue. The study also showed that the protein promotes autophagy [109]. 

Another study conducted on SGC-7901 and MGC-803 cancer cell lines with SIRT5 overexpression showed that SIRT5 inhibits GC cell proliferation, the ability to form colonies, and aerobic glycolysis in vitro. Tang et al. [83] examined tumor growth and promotion ability in vivo. Nude mice were injected with SGC-7901 line cells with SIRT5 overexpression and then the size of the formed tumors was examined. The measurement of the growth curve demonstrated that SIRT5 overexpression inhibited the volume and mass of tumors formed from SGC-7901 cells. SIRT5 overexpression also facilitated apoptosis in SGC-7901 and MGC-803 GC cells [83]. 

### 4.6. Sirtuin 6

Sirtuin 6 (SIRT6) is involved in fatty acid metabolism, influences the secretion of TNF, a cytokine, that plays a key role in cancer pathogenesis, as well as modulates NF-κB-related metabolic pathways [110]. SIRT6 can act both as the promotor and suppressor of carcinogenesis. Human SIRT6 is found in the nucleus and is engaged in a range of enzymatic interactions. SIRT6 was shown to display deacetylase activity on histone H3 that modulates telomeric chromatin at pericentric regions and thus controls the expression of many glycolytic genes. More importantly, SIRT6 can act as transcriptional repressor and maintains genome stability. SIRT6 interacts with several elements of DNA repair machinery. It is involved in the repair of DNA double-strand breaks (DSB) through the deacetylation of C-terminal binding protein (CtBP), C-terminal interacting protein (CtIP), PARP-1 (Poly [ADP-ribose] polymerase 1) and DNA-dependent protein kinase (DNA—PK). The presence of fatty acids induces SIRT6, which removes long-chain fatty acids from other proteins, such as TNFα. It is also a corepressor of MYC activity in ribosomal genes [111].

A study conducted by Zhou et al. [84] indicates that SIRT6 inhibits GC growth by inactivating the JAK2/STAT3 signaling pathway. The analysis of clinical research showed that patients with lower SIRT6 levels tend to have larger tumor sizes and more advanced cancer staging. Moreover, the colony formation test showed that SIRT6 overexpression significantly inhibited colony formation by GC cells, in addition to their motility and proliferation [84]. 

### 4.7. Sirtuin 7

Sirtuin 7 (SIRT7) is the latest discovered and least studied sirtuin [112], and it is mainly involved in the deacetylation of lysine residues at the K18 position of histone H3 (H3K18Ac) [113]. Like other sirtuins, it is involved in the regulation of many metabolic processes. SIRT7 is located in the nucleolus, where it enables rDNA transcription through the deacetylation and activation of Pol I subunit PAF53. SIRT7 is involved in the processing of pre-rRNA through the deacetylation and activation of U3-55k (the core component of small nucleolar ribonucleoproteins (snoRNPs)). Zhang et al. [63] found that the level of SIRT7 was substantially higher in GC cells compared to healthy gastric epithelium cells. They also found that SIRT7 expression was clearly correlated with tumor size, metastasized cancer, advanced stage and poor prognosis. High SIRT7 expression was associated with poor survival. SIRT7 knockdown reduced GC growth in in vitro and in vivo tests. SIRT7 prevents cell apoptosis through miR-34a downregulation owing to H3K18ac deacetylation. The novel Sirt7/miR-34a regulatory pathway identified by Zhang et al. casts a new light on gastric carcinogenesis. The restoration of miR-34a expression or SIRT7 knockdown can be a potential therapeutic strategy in the treatment of GC. SIRT7 also boosts tRNA transcription via Pol III and inhibits ELK4 and MYC transcription, thus promoting tumor growth. What is important is that SIRT7 also inhibits p53 protein activity. In the conducted research, SIRT7 knockdown in GC cells inhibited cell proliferation and colony formation in vitro. Additionally, the loss of SIRT7 induced apoptosis of GC cells through the increased expression of pro-apoptotic proteins and inhibited the expression of anti-apoptotic proteins [63].

## 5. Regulation of SIRTs As a Potential Target in GC Therapy

Encouraged by these results, many scientists have made efforts to study the therapeutic potential of SIRT (especially SIRT1) activators or inhibitors, natural and synthetic small molecules included in clinical practice. The deacetylase of SIRT can be modulated by cellular stress (fasting, glucose & calorie restriction), some protein factors (AROS, SUMO, NAD^+^/NADH, etc.), by agonists (resveratrol, SRT1720), or by inhibitors (tenovins, sirtinol) [97].

In this review we describe the crucial modulating factors for SIRTs, making them the potential target in GC therapy.

### 5.1. Resveratrol

Resveratrol (RSV) is the best known and most studied activator of SIRTs, especially SIRT1. It is a natural polyphenolic flavonoid that has been considered as an antioxidant drug in various autoimmune inflammatory diseases, as well as in cancer therapy. RSV improves cell sensitivity to insulin and also blood vessel function [48]. The resveratrol-induced activation of SIRT1 has great therapeutic potential and gives satisfactory results in the treatment of patients, by acting as anti-inflammatory, antiangiogenic, antioxidant, proapoptotic, antiaging and anticancer [114,115] agent. This is crucial in the context of gastric cancer pathogenesis, as resveratrol was found to also have antimicrobial effects through the inhibition of the growth of multiple *H. pylori* strains [116,117].

The anti-tumor activities of resveratrol were reported for the first time in 1997 [118]. Furher reports have shown that resveratrol can suppress the proliferation of several types of cancers, e.g., breast [119], pancreas [120], prostate [121], and colon [122], and affects diverse molecular targets (SIRT, EGF, *H. pylori*). It was shown that a diet rich in resveratrol exhibits both apoptosis and autophagy-promoting activities in GC cells [123]. Similarly, Yang et al. [69] found that resveratrol inhibited the proliferation of the GC cell lines AGS, BGC-823 and SGC-7901, inducing senescence instead of apoptosis. By inhibiting autophagy it could enhance the antitumor effects of these drugs in the treat-ment of GC, suggesting that autophagy plays a protective role against GC cells from death [124].

Wang et al. [125] was treating human gastric adenocarcinoma SGC7901 with resveratrol for 48 h in order to determine the role of reactive oxygen species (ROS) and sirtuin1 in resveratrol-induced cellular apoptosis. Indeed, after 2 days, resveratrol at the appropriate dose (50–200 μmol/L) significantly induced apoptosis and DNA damage in human gastric cancer cells. The data shows evidence that resveratrol induces apoptosis via ROS, but independent of SIRT1 [125]. Yang et al. [69] also found that resveratrol inhibited the proliferation of GC cells in a dose-dependent manner. GC cells were arrested in the G1 phase and this led to senescence instead of apoptosis. They also evaluated the effects of RSV in vivo by using a nude mice xenograft model with GC. Data showed that resveratrol inhibits GC in a SIRT1-dependent manner and positively affects in GC prevention and therapy. The chemo preventive effects of RSV were shown by Buhrmann et al. [126] in colorectal cancer cells. They found that resveratrol suppressed the proliferation of HTC116 and SW480 cancer cells, downregulated NF-κB phosphorylation and acetylation, and reduced the amount of NF-κB-regulated gene products, which are important for tumor invasion and metastasis (MMP-9, CXCR4). The next step was to discover that the effect of resveratrol can be potentiated by combinatorial treatment of resveratrol and inhibitors, such as cytochalasin D (CytD) and focal adhesion kinase-inhibitor (FAK-I) [127]. It is known that during tumor formation neoplastic cells can come into contact with TME cells. As was shown by Buhrmann et al. [128], resveratrol can suppresses this cross-talk between colorectal cancer cells and stromal cells in TME. The RSV/SIRT1 axis suppresses this communication by modulating NF-κB signaling and by paracrine agent secretion. The authors also recall that fibroblasts and T cells can also be promising targets in CRC metastasis, controlled by resveratrol.

Information about other substances, both natural and synthetic, in the regulation of SIRT is negligible. We have collected the available data and presented them in Table 2.

### 5.2. Dietary Restriction

The restriction of nutrients and energy, also known as caloric restriction, or fasting (in a more restrictive modelis), is one of the actions that regulates the activity of sirtuins, proteins that are responsible for cell metabolism The relationship between caloric intake, body weight and cancer risk has been confirmed [129]. An unreasonable and unhealthy diet is mentioned as one of the causes of gastric cancer [130,131,132,133].

As in every aspect, we know the most about SIRT1 activation by fasting, caloric restriction, or adopting a vegetarian diet. The activation of SIRT1 by calorie restriction stimulates PGC-1α and reduces the repressive effect of PGC-1α on glycolytic genes, thus increasing hepatic glucose output. By activating PPAR-α, SIRT1 increases fatty acid oxidation. As shown in a group of gastric cancer patients, a very low-calorie diet program (lasting 20 days) has positive effects before gastric cancer laparoscopic gastrectomy [133]. Even parenteral nutrition with low-nitrogen and a low-calorie diet combined with enteral nutrition can effectively reduce the inflammatory reactions and improve the immune function, quality of life and even prognosis in patients with gastric cancer [134]. The ketogenic diet (KD) is based on limiting the access of carbohydrates (to max. 5–10% of total daily caloric intake), which causes the metabolism to shift towards ketone bodies. There are limited data about the influence of KD on gastric cancer cells. Otto et al. have shown that the growth of human gastric cancer cells in nude mice is retarded by a ketogenic diet supplemented with omega-3 fatty acids [135], but further studies are needed to define the KD impact on GC metastasis. The beneficial effects of fasting, CR or vegetarian diets in gastric cancer patients are confirmed, therefore it is worth considering it in the treatment plan.

## 6. Conclusions

Chronic inflammation is not exclusive to autoimmune diseases, but is a general feature of most common diseases of aging, including cancer. As was presented in this narrative review, sirtuins can participate in several steps of the gastric cancer pathogenesis process, including responding to *H. pylori* infection, tumor formation, growth, angiogenesis, apoptosis inhibition, proliferation and migration. We presented the relevant data about the role of all SIRTs in GC pathogenesis. SIRT1 as an epigenetic regulator in response to DNA damage is the best described sirtuin, but the roles of the six other sirtuins (SIRT2–7) are also significant. Importantly, the research cited in this review often sees sirtuins as a potential biomarkers or therapeutic targets [136]. Thus, molecules and substances that have strong antioxidant potential and affect the SIRTs, or caloric restriction and fasting, can potentially help protect patients against GC initiation and progression. It is important to find an effective cancer treatment with minimal side effects. Patients are often advised to use caloric restriction/ fasting prior to chemo-and radiotherapy as a supportive therapy. The available data show that sirtuins may be a real target of anti-cancer therapy in the future, especially those SIRT with a dual-role, but many years of research is required to confirm this.

## Figures and Tables

**Table 1:** The role of sirtuins in gastric cancer.

Name	Function	References
SIRT1	inhibits GC cell proliferation and tumor growth; GC growth and metastasis by FOXO1 and YAP signaling; participates in mitophagy; deacetylation of histone substrates, transcription factors and cofactors (p53, STAT3, DBC1, FOXO, c-Myc & Ku70); RSV prevents STAT3 and NF-κB activation;	[64,67,74,75,76,77]
SIRT2	influences the migration and invasion of GC cells to metastatic niche;	[62]
SIRT3	inhibition of NOTCH1 expression; blocking SIRT3 expression promoted cell division and tumor growth; decreased HIF-1α and ROS production; promoting proliferation, glucose uptake, MnSOD activity;	[78,79,80,81]
SIRT4	inhibiting cell proliferation, migration, and invasion; reduces the number of colonies formed by GC cells; stops the cell cycle in the G1 phase;	[65,82]
SIRT5	promotes autophagy; reduces the number of colonies and the viability of GC cells;	[83]
SIRT6	inhibits cell viability, proliferation, colony formation, and cell cycle; increases apoptosis; inhibits the JAK2/STAT3 pathway;	[84]
SIRT7	promotes GC cells proliferation and growth; cell survival and migration; inhibits apoptosis;	[30,63]

**Table 2:** The role of sirtuins in GC and known factors regulating their activity.

Name	Modulated By
SIRT1	natural: resveratrol, quercetin, apigenin, catechin, epicatechin, theobromine, curcumin, soy isoflavones, sulforaphane, olivetol, isothiocyanates, piceatannol, cinnamon;
	synthetic: S17834, SRT1720;
SIRT2	synthetic: tempol, (SOD1), 1,4-DHP derivative;
SIRT3	natural: resveratrol, epicatechin, curcumin, piceatannol;
	synthetic: acetyl-CPS1,1,4-DHP derivative;
SIRT4	natural: resveratrol, curcumin;
	synthetic: DMS-CPS, HMG-CPS1, acetyl-CPS1;
SIRT5	snatural: piceatannol;
	synthetic: succinyl peptide, DMS-CPS, HMG-CPS1, UBCS039;
SIRT6	natural: iso-quercetin, luteolin, and cyanidin;
	synthetic: tempol, copper-zinc superoxide dismutase (SOD1) mimetic, pyrrole (1,2a) quinoxaline derivative, UBCS039;

## Data Availability

Not applicable.
